# Fat mass and obesity associated (FTO)-mediated N6-methyladenosine modification of Krüppel-like factor 3 (KLF3) promotes osteosarcoma progression

**DOI:** 10.1080/21655979.2022.2051785

**Published:** 2022-03-21

**Authors:** Hong-Jian Shan, Wen-Xiang Gu, Gang Duan, Hong-Liang Chen

**Affiliations:** a Department of Orthopedics, Institute of Orthopedics, the Affiliated Hospital of Xuzhou Medical University, Xuzhou, Jiangsu, P. R. China; b Department of Orthopedics, the Affiliated Jiangning Hospital with Nanjing Medical University, Nanjing, Jiangsu, 211100, P. R. China; c Department of Orthopedics, the Second Affiliated Hospital of Xuzhou Medical University, Xuzhou, Jiangsu, 221002, P. R. China

**Keywords:** m6A methylation, FTO, osteosarcoma, KLF3

## Abstract

N6-methyladenosine (m6A) methylation is the most common and abundant methylation modification of eukaryotic mRNAs, which is involved in tumor initiation and progression. The study aims to explore the potential role and the regulatory mechanism of fat mass and obesity associated (FTO) in osteosarcoma (OS) progression. In this study, we detected the expressions of Krüppel-like factor 3 (KLF3) in OS cells and tissues and found that the mRNA and protein levels of KLF3 were increased in OS cells and tissues and significantly related to tumor size, metastasis, and TNM stage and poor prognosis of OS patients. FTO promoted the proliferation and invasion and suppressed apoptosis of OS cells through cell experiments in vitro. Further mechanism dissection revealed that FTO and YTHDF2 enforced the decay of KLF3 mRNA and decreased its expression. FTO-mediated mRNA demethylation inhibited KLF3 expression in the YTHDF2-dependent manner. Moreover, KLF3 overexpression abrogated FTO-induced oncogenic effects on the proliferation and invasion of OS cells. Overall, our findings showed that FTO-mediated m6A modification of KLF3 promoted OS progression, which may provide a therapeutic target for OS.

## GRAPHICAL ABSTRACT

**Figure:**
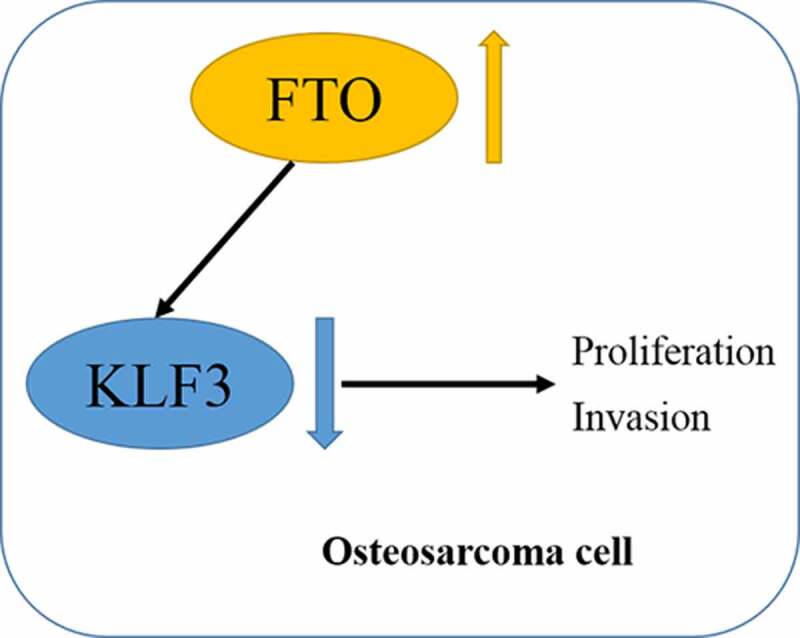

## 1. Introduction

Osteosarcoma (OS), as the most common primary malignant bone tumor, originates from mesenchymal cells, mostly in long bone, and can also be seen in distal femur or proximal tibia. OS is often accompanied by micrometastasis before operation and is characterized by recurrent possibility and suboptimal prognosis [1]. Approximately 15%-20% of patients with OS are diagnosed with distant metastases at the first diagnosis [2]. Even after radiotherapy and chemotherapy, the survival rate of 5-year is still less than 20% for OS complicated with lung metastasis [3]. The combination of surgery and radiotherapy or chemotherapy has made some progress in improving the prognosis of patients with OS, but the curative effect is still not ideal [4]. Therefore, we urgently need to explore the mechanism of OS tumorigenesis, identify key molecular targets and improve the prognosis of OS.

m6A methylation is a dynamic and reversible epigenetic modification of RNAs, which mainly affects the splicing, translation, and stability of mRNAs and the epigenetic effects of non-coding RNAs [5,6]. The biological processes of m6A modification are mainly regulated by three kinds of key-related kinases, m6A methyltransferase (Writers), m6A demethylase (Erasers), and methylation reading protein (Readers) [5,6]. Methyltransferase complexes composed of various writers, such as METTL3/14, WTAP, and KIAA1429, catalyze the m6A modification of adenylate on mRNAs. Demethylases including fat mass and obesity associated (FTO) and ALKHB5 catalyze the m6A demethylation. The main function of readers is to recognize the m6A-modified RNAs, thus activating downstream regulatory pathways such as RNA processing, mRNA degradation, translation, and decay [7,8]. m6A RNA methylation plays multiple roles in mammalian, including growth and development, embryonic development, circadian rhythm, and DNA damage response. Accumulating evidence suggests that m6A methylation is closely related to tumorigenesis and progression [6,9,10]. METTL3 promotes cell proliferation, migration, and invasion and activates the Wnt/β-catenin pathway in OS through regulating m6A methylation of LEF1 [11]. Blockade of METTL3 inversely inhibits growth and invasion of OS cells by suppressing ATAD2 [12]. ALKBH5 augments lncRNA PVT1 expression in an m6A-dependent manner and facilitates OS tumorigenesis [13]. These studies show that m6A methylation plays an important role in the occurrence and progression of OS, but the specific mechanism and role of m6A reader FTO has not been reported.

In 2011, FTO was the first identified RNA-demethylase, revealing that m6A methylation is dynamic and reversible epigenetic modification [14]. The *FTO* gene is located on chromosome 16q12.2, with 9 exons, and it is widely expressed in different stages of human development, especially in the brain [15]. FTO, as an α-ketoglutarate acid- and Fe^2+^-dependent non-heme dioxygenase belonging to the ALKB family, reversibly catalyzes the m6A demethylation [16]. FTO originally considered as a susceptible gene plays an important role in lipid metabolism and accumulation and obesity [16]. Increasing evidence has emerged to reveal the complex biological functions of FTO, especially tumor initiation and progression [17]. The pathological role of FTO as an m6A demethylase in cancer was first reported in acute myeloid leukemia, and FTO inhibitor R-2-hydroxyglutarate exerts tumor-suppressive activity by targeting FTO/C-Myc/CEBPA pathway, revealing great clinical value [18]. Both up-or down-regulated FTO has been implicated in carcinogenesis and tumor progression [17]. However, the potential of FTO in OS progression remains undefined.

Krüppel-like factor 3 (KLF3), a member of the KLF family, was originally a KLFI homolog cloned from human hematopoietic tissue. KLF3 can recruit transcriptional corepressor C-terminal binding protein and complete fusion in the target gene promoter, regulating transcription of target genes [19,20]. KLF3 plays important roles in B cell development, erythrocyte and fat production, nervous system and muscle gene regulation, cell differentiation, and proliferation [19,20]. Aberrant expression of KLF3 has been detected in several cancers, such as lung cancer [21], acute leukemia [22], colorectal cancer [23], soft tissue sarcoma [24]. Silencing KLF3 contributes to lung cancer metastasis and the EMT process by regulating the STAT3 signaling pathway [21]. Decreased KLF3 is associated with aggressive phenotypes and poor prognosis for colorectal cancer patients [23]. KLF3 inhibits the pro-metastatic miR-182 expression and human soft tissue sarcoma metastasis [24]. All these support the potential tumor-suppressor role of KLF3. However, the underlying mechanism by which KLF3 is affected, especially m6A methylation, is virtually unknown in OS.

The present study aimed to investigate the expression profile of FTO in OS and its underlying mechanisms in OS progression. We observed that FTO was increased expression and related to a poor prognosis of OS patients. We hypothesized that FTO elicited potent oncogenic functions in OS development. Moreover, KLF3 was identified as a potential downstream target of FTO. Overall, our data provided a theoretical basis for FTO in OS progression by regulating KLF3.

## 2. Methods and methods

### 2.1 Tissue samples

A total of 42 pairs of primary OS and adjacent tissues are collected from the Affiliated Hospital of Xuzhou Medical University. All patients with OS did not receive any radiotherapy or chemotherapy before the operation. All cases were confirmed by experienced pathologists, and clinical information is fully recorded (Supplementary Table 1). All patients provide written informed consent, and the study involving human specimens is approved by the Review Board of the Affiliated Hospital of Xuzhou Medical University.

### 2.2 Cell culture and transfection

Human OS cell lines (HOS, MG63, SaOS2, U2OS) and a normal osteoblast Nhost cell line were acquired from the Chinese Cell Bank of the Chinese Academy of Sciences (Shanghai, China). Cells were cultured in DMEM (Gibco, Grand Island, USA) supplemented with 10% fetal bovine serum (FBS; Gibco, Grand Island, USA), 100 U/mL penicillin, and 0.1 mg/mL streptomycin (Vicmed, Xuzhou, China). All cells were maintained at 37°C with 5% CO_2_.

The plasmids of cDNAs expressing FTO, KLF3, and YTHDF1 and negative control (NC) were transiently transfected into OS cells by using Lipofectamine 2000 (Invitrogen, USA) following the manufacturer’s protocol. siRNAs (siFTO and siYTHDF2) and negative control (siNC) were transfected using Lipofectamine 2000 (Invitrogen, USA). siRNA sequences are as follows (5’ to 3’) [25]: siNC, 5’-UUCUCCGAACGUGUCACGUTT-3’; siFTO, 5’-TTAAGGTCCACTTCATCATCGCAGG-3’, and siYTHDF2, 5’-AAGGACGTTCCCAATAGCCAA-3’. After 24 h or 48 h transfection, cells were harvested.

### 2.3 Western blotting

After transfection, cells were collected and lysed with radioimmunoprecipitation assay (RIPA) buffer (Beyotime Biotechnology, Shanghai, China) containing proteinase inhibitor PMSF (Vicmed, Xuzhou, China). Protein concentration was measured using a bicinchoninic acid (BCA) protein assay kit (Beyotime, Shanghai, China). The protein sample was separated by 10% SDS-PAGE and transferred onto the nitrocellulose membrane with 0.2 µm of pore (PALL, Port Washington, NY, USA). The membrane was blocked with 5% nonfat milk for 2 h and incubated overnight at 4°C with primary antibodies: FTO (27226-1-AP, Proteintech, Wuhan, China), YTHDF2 (24744-1-AP, Proteintech), GAPDH (10494-1-AP, Proteintech), N6-methyladenosine (ab208577, Abcam, Cambridge, MA, USA), KLF3 (ab154531, Abcam), p21 (ab109520, Abcam), Cyclin D1 (ab16663, Abcam), N-cadherin (ab76011, Abcam), E-cadherin (EM0502, HUABIO, Hangzhou, China). The membrane was then probed with horseradish peroxidase (HRP)-conjugated secondary antibodies (Anti-rabbit or moues; SA00001-2 or SA00001-1, Proteintech) at room temperature for 2 h. The protein band was visualized by ECL Western Blotting Substrate (Tanon, Shanghai, China) using the Chemiluminescence imaging analysis system (Tanon).

### 2.4 Quantitative real-time PCR (qPCR)

TRIzol Reagent (Vazyme, Nanjing, China) was employed to extract total cellular RNA from tissues or cells following the manufacturer’s protocol. PrimeScript RT reagent Kit (Servicebio, Wuhan, China) was used for cDNA synthesis. qPCR analysis was performed using the SYBR Green PCR Kit (Servicebio) with the ABI Step-One Plus^TM^ Real-Time PCR System (Applied Biosystems). All results were normalized to GAPDH. The relative expression of mRNAs was quantified using the 2^−ΔΔCt^ method. Primer sequences used for qPCR were as follows: FTO forward 5’- ACTTGGCTCCCTTATCTGACC-3’ and reverse 5’-TGTGCAGTGTGAGAAAGGCTT-3’, GAPDH forward 5’-GTCTCCTCTGACTTCAACAGCG-3’ and reverse 5’-ACCACCCTGTTGCTGTAGCCAA-3’, KLF3 forward 5’- TGTCTCAGTGTCATACCCATCT-3’ and reverse 5’-CCTTCTGGGGTCTGAAAGAACTT-3’, and YTHDF2 forward 5’-AGCCCCACTTCCTACCAGATG-3’ and reverse 5’-TGAGAACTGTTATTTCCCCATGC-3’.

### 2.5 Immunohistochemistry (IHC) assay

IHC analysis was performed as previously reported [26]. Briefly, the formalin-fixed, paraffin-embedded tissue sections were deparaffinized in xylene, rehydrated in gradient ethanol. Microwave heat-induced antigen retrieval was performed in Citrate Buffer (0.01 M) solution for 10 min. Endogenous peroxidase activity was blocked with 30% hydrogen peroxide for 15 min. After blocking with 10% normal goat serum for 1 h at room temperature, the section was incubated overnight at 4°C with the primary antibodies, FTO (27226-1-AP, Proteintech) and KLF3 (ab154531, Abcam), followed by the appropriate secondary antibodies. Immunopositive staining was visualized by the treatment with 3, 3’-diaminobenzidine (Zhongshan biotech, Beijing, China). The IHC staining was evaluated based on the staining intensity and the positive percentage of each section. The staining intensity was divided into negative (0), weak (1), moderate (2), and strong (3), and the positive percentage was divided into 0–25% (1), 26–50% (2), 51–75% (3), and 76–100% (4). Finally, the immunoreactive score of FTO was the product of positive percentage and staining intensity. And FTO was divided into low (score < 6) and high (score ≥ 6) expression in OS tissues (Supplementary Table 1).

### 2.6 Cell proliferation assay

Cell proliferation assay was performed using Cell Counting Kit-8 (CCK-8; Vicmed, Xuzhou, China). In brief, transfected OS cells (2 × 10^3^ cells/well) were plated in 96-well plates and cultured at 37°C for 24 h, 48 h, and 72 h. Then, 10 µl of cell CCK-8 solution was added to each well for incubation for 2 h. Cell proliferation was detected by measuring the absorbance at 450 nm using a microplate reader. The experiment was performed in triplicate.

### 2.7 Invasion assay

Cell invasion assay was performed using transwell chambers containing 8-mm pores (Corning, USA). The upper chamber membrane was coated with Matrigel (BD Bioscience, USA). Then 200 μL of cell suspension (2 × 10^5^ cells) with serum-free was inoculated into the top compartment of the chamber, and 600 μl of 10% FBS-containing medium was added into the lower chamber as an attractant. After the incubation for 24 h, non-invaded OS cells were removed from the top chamber membrane using a cotton swab, and the invasive cells on the bottom of the chamber were fixed with 4% paraformaldehyde for 10 min, stained with 0.1% crystal violet, and photographed using a microscope. The experiment was performed in triplicate.

### 2.8 Wound healing assay

Wound healing assay was performed to evaluate the migration ability of OS cells. Transfected OS cells were seeded in 6-well plates. Cell monolayer was scraped using a sterile 200-µL pipette tip to form artificial wounds when cells reached a density of 70–80%. Cells were washed 3 times to remove the cell fragments and then cultured with serum-free medium for 24 h. cell wound was imaged using a microscope at 0 h and 24 h. The percentage of wound healing was determined through the formula: (1-[24-h wound size/0-h wound size]) × 100%.

### 2.9 Flow cytometry-based apoptosis detection

Fluorescein Annexin V-FITC/PI double labeling was performed using Annexin V-FITC/PI Apoptosis Detection Kit (Elabscience Biotechnology, Wuhan, China) to detect the apoptotic cells according to the manufacturer’s instructions. Transfected OS cells were collected and washed 3 times with PBS. Cells were then re-suspended in 500 μL of 1 × Annexin V Binding Buffer diluted by deionized water, followed by the treatment with the 5 μL of Annexin V-FITC and 5 μL of PI staining Buffer. Finally, the apoptotic cells were determined with a flow cytometer (BD Biosciences, US) and analyzed with FlowJo 10 Software (FlowJo Systems, Tree Star, OR).

### 2.10 m6A quantification assay

m6A quantification assay was performed using the m6A RNA Methylation Quantification Kit (Colorimetric) (Abcam, ab185912) following the manufacturer’s instructions. Total RNA was extracted with TRIzol Reagent (Vazyme). Briefly, 80 µL of Binding Solution was added to each well, then 200 ng of RNA sample and 2 µL of Positive and Negative Control was also added to the wells. After the incubation at 37°C for 90 min, each well was added 50 µL of the Diluted Capture Antibody and then incubated for 60 min at room temperature. Each well was successively added 50 µL of the Diluted Detection Antibody and 50 µL of the Diluted Enhancer Solution. For the signal detection, each well was treated with 100 µL of Developer Solution and Stop Solution, and the absorbance at 450 nm was detected by using a microplate reader. The percentage of m6A in total RNA was calculated via the formulae: ([(Sample OD – NC OD) ÷ S] ÷ [(PC OD – NC OD) ÷ P]) × 100%. S, input sample RNA; P, input Positive Control.

### 2.11 Dot blot assay

Total RNA isolated from OS cells was mixed in three times volume of incubation buffer and denatured at 65°C for 5 min, and chilling directly on ice, 200 ng, 400 ng, and 800 ng of samples mixed with the ice-cold SSC buffer (Sangon Biotech, China) were spotted to a nitrocellulose filter membrane. After the membrane was dried, it was subjected to the UV cross-linking for 10 min and washed 3 times with PBST. Then, it was stained with 0.02% Methylene blue (Solarbio, Beijing, China) as a loading control. After blocking with 5% milk for 1 h, the membrane was incubated with m6A antibody (ab208577, Abcam) overnight at 4°C. Finally, the membrane was incubated with the HRP-conjugated anti-rabbit immunoglobin IgG secondary antibody for 2 h before the visualization by ECL Western Blotting Substrate (Tanon, Shanghai, China).

### 2.12 RNA stability assay

Transfected OS cells were incubated with the transcriptional inhibitor Actinomycin D (5 μg/mL) at 0 h, 3 h, 6 h, 9 h. The total RNA from the cells was isolated by TRIzol Reagent (Vazyme). The remaining expression of KLF3 mRNA for each group was calculated using qPCR.

### 2.13 RNA immunoprecipitation (RIP) assay

RIP assay was performed using the Magna RIP™ RNA-binding protein immunoprecipitation kit (Millipore, Catalog No. 17–700, Bedford, MA, USA) according to the manufacturer’s instruction. Briefly, cells were harvested using the RIP Lysis Buffer supplemented with RNase inhibitor and protease inhibitor Cocktail. Magnetic beads were pre-coated with the YTHDF2 (24744-1-AP, Proteintech) or IgG negative control antibodies at 4°C overnight with rotation. After discarding the supernatant, RIP Immunoprecipitation Buffer and cell lysis sample were added to each tube, followed by incubation overnight at 4°C with rotation. The following day, immunoprecipitated RNA was purified with proteinase K buffer supplemented with 10% SDS, proteinase K, and RIP Wash Buffer for the incubation at 55°C for 30 min. Finally, immunoprecipitated RNA was extracted using the mixture solution of phenol: chloroform: isoamyl alcohol and reverse-transcribed and detected by qPCR.

### 2.14 Methylated-RNA immune precipitation (Me-RIP) assay

Me-RIP assay was carried out using Magna Me-RIP^TM^ m6A Kit (Millipore) according to the standard protocol. Briefly, total RNAs were extracted with TRIzol Reagent (Vazyme) and the mRNA was purified using GenEluteTM mRNA Miniprep Kit (Sigma, Louis, MO). Then the mRNA was subjected to fragmentation into approximately 100-nt fragments using ZnCl_2_ and then incubated with anti-m6A antibody for m6A immunoprecipitation overnight at 4°C with rotation. The immunoprecipitation mixtures of antibody-RNA were captured using Magnetic beads via incubation for 4 h at 4°C. The m6A enriched RNAs were eluted from the Magnetic beads and subjected to ethanol precipitation. Finally, the enrichment of m6A containing mRNA was analyzed by qPCR and calculated by normalizing to the input. The specific sequences of KLF3 for Me-RIP assay were as follows: forward 5’-GCACATGGAAGTTTGCTCGG-3’ and reverse 5’-AGGGCAAGATGGTCAGAACG-3’.

### 2.15 Statistical analysis

All data were presented as the mean ± SD from three independent experiments. Unpaired Student’s t tests and one-way ANOVA were used for two groups and multiple comparisons, respectively. Chi-square was applied to evaluate the relationship between FTO expression and OS clinicopathological features. Survival curves were generated with the Kaplan-Meier method and log-rank test. Correlation analysis FTO and KLF3 mRNA expressions in OS tissues were evaluated by the Pearson correlation test. Two-sided *p* values < 0.05 were considered statistically significant.

## 3. Results

In the study, we aimed to explore the expression of FTO in OS and found that FTO was upregulated in OS and was associated with a poor prognosis of OS patients. A series of assays confirmed that FTO promoted the proliferation and migration of OS cells by regulating KLF3 expression via an m6A-dependent manner. Our findings highlight the regulatory function of FTO in OS and provide new ideas for OS pathogenesis.

### 3.1. FTO is increased expression in OS

**Figure 1.**
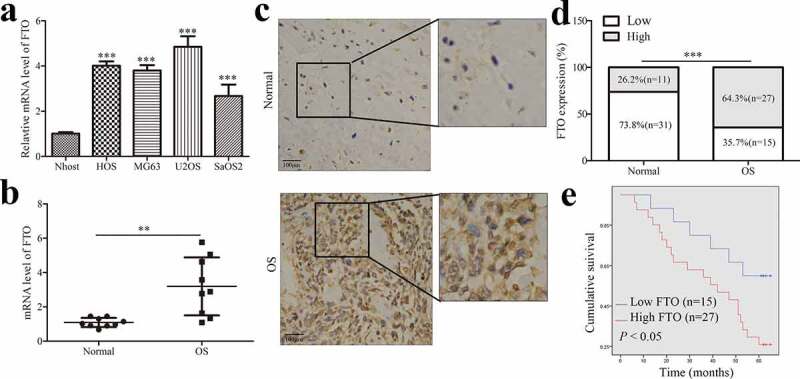
FTO is increased in OS tissues.

To explore the potential role of FTO, we examined the FTO expression in OS cells and normal osteoblast Nhost cells by qPCR. Results showed that the mRNA levels of FTO in OS cells were elevated (Figure 1a). We also detected the mRNA levels of FTO in OS and adjacent tissues. Similarly, mRNA expression of FTO was frequently increased in cancer tissues compared with matched normal tissues (Figure 1b). IHC staining was also used to detect the expression pattern of FTO in OS and matched normal tissues (Figure 1c). According to the quantitative analysis of IHC staining of tissue samples, we divided the FTO expression into high expression and low expression. We found that 64.3% of OS tissues showed high expression, whereas the low expression rate of FTO in normal tissues was only 26.2% (Figure 1d), implying the potential role of FTO in OS tumorigenesis.

The mRNA levels of FTO were detected in OS cells and normal osteoblast Nhost cells (a). The mRNA levels of FTO in 9 paired OS tissues and the corresponding normal tissues (b). Representative images of IHC staining were used to analyze the protein levels of FTO in OS tissues adjacent and normal tissues (c). FTO expression was calculated in OS and normal tissues via quantitative analysis of IHC staining of FTO protein (d). Kaplan–Meier and log-rank test were used to analyze the 5-year overall survival OS patients with high or low FTO expression (e). Data represent the means ± SD. **, *P* < 0.01; ***, *P* < 0.001.

We further analyzed the correlations between the FTO expression and clinicopathological parameters and postoperative survival of OS. IHC assay indicated that there were significant correlations between FTO expression and several clinicopathological features (Supplementary Table 2), such as tumor size, metastasis, and TNM stage, but not with gender and age. To further understand the clinical significance, we performed a Kaplan-Meier survival curve analysis according to the FTO expression. The FTO expression was negatively correlated with the 5-year overall survival of OS patients (Figure 1e), which suggested that FTO might serve as a prognostic factor in OS patients.

### 3.2. FTO promotes OS cell proliferation, migration and invasion

**Figure 2.**
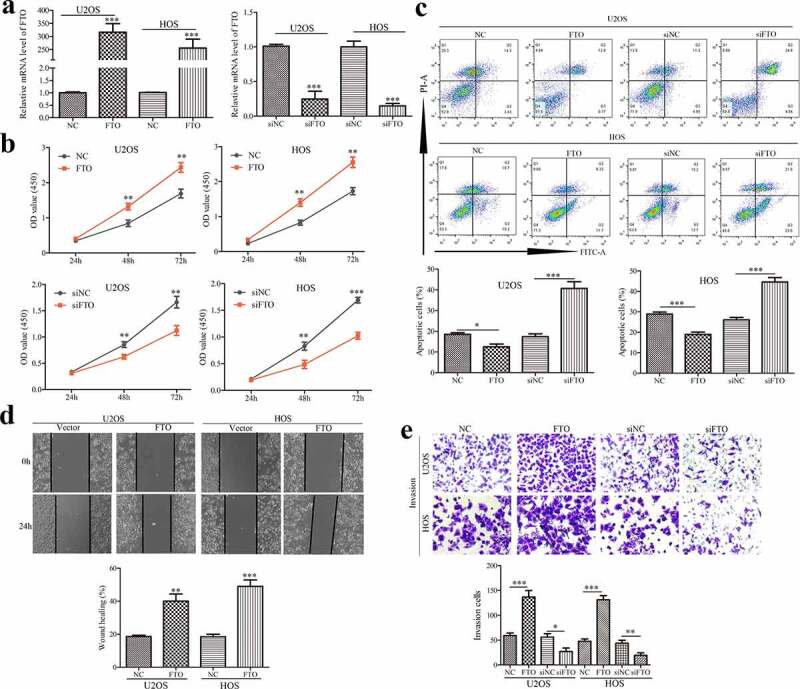
FTO promotes proliferation, invasion of OS cells and inhibits cell apoptosis.

To further explore the effect of FTO on the proliferation of OS cells, FTO knockdown or overexpression was used to detect the proliferation ability of OS cells at 24 h, 48 h, 72 h, respectively. Transfection efficiencies of FTO knockdown or overexpression were confirmed by qPCR in OS cells (Figure 2a). Results from CCK-8 assays showed that overexpression of FTO in OS cells promoted cell proliferation (Figure 2b), while knockdown of FTO inhibited cell proliferation (Figure 2b). As shown in (Figure 2c), enforced expression of FTO inhibited OS cell apoptosis, however, blockade of FTO expedited apoptosis.

Transfection efficiencies of FTO knockdown or overexpression were analyzed by qPCR in OS cells (a). The cell viability of OS cells was measured by CCK8 assay after FTO knockdown or overexpression (b). FTO-knockdown or -overexpression on cell apoptosis was determined by flow cytometry assays. Q1, necrotic; Q2, late apoptotic; Q3, early apoptotic; Q4, live. (c). OS cell motilities were analyzed by measuring the wound closure after FTO knockdown or overexpression (d). Transwell chamber assays were performed to detect the invasive ability of OS cells after FTO knockdown or overexpression (e). Data represent the means ± SD. *, *P* < 0.05; **, *P* < 0.01; ***, *P* < 0.001.

Wound healing assay was used to verify the migration ability of FTO. Amplification of FTO accelerated scratch healing of OS cells (Figure 2d). Transwell chamber assays showed that FTO facilitated the invasion of OS cells; inversely, silencing FTO attenuated the invasive ability (Figure 2e). These findings indicated FTO promoted cell proliferation, migration, and invasion, which supported a potent oncogenic effect of FTO on OS.

### 3.3. FTO inhibits KLF3 expression in OS

**Figure 3.**
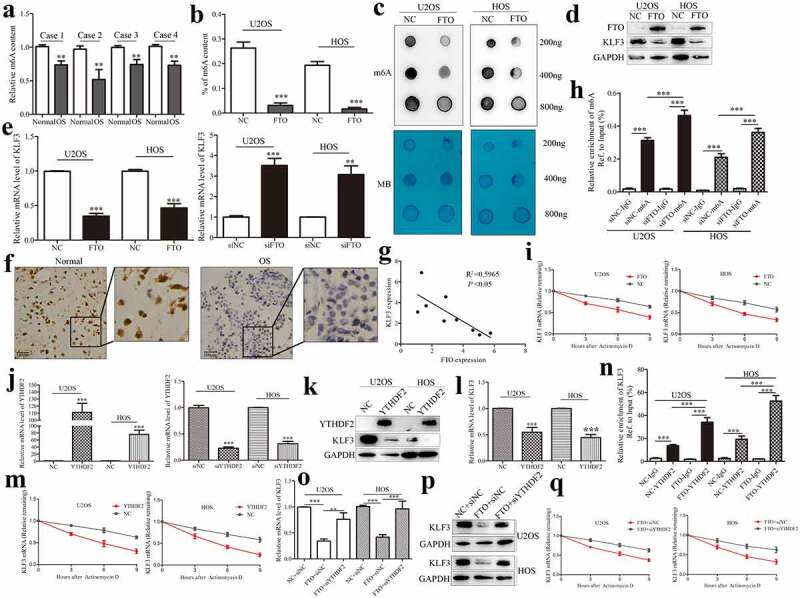
FTO regulates KLF3 expression in an m6A-dependent manner in OS cells.

As the first discovered demethylase FTO, its discovery reveals the dynamic and reversible process of m6A modification, and it can reverse the m6A modification on RNAs [27]. Previous results indicated that KLF3 is decreased expression in human soft tissue sarcomas [24]. It has been reported that the tumor suppressor KLF4, another member of the KLF family, could be regulated in an m6A-dependent manner in bladder cancer [28]. METTL3-mediated m6A methylation directly promotes the mRNA decay of KLF4 through m6A reading protein YTHDF2 [28]. Similarly, we identify whether the role of KLF3 in OS is regulated by m6A eraser FTO. We subsequently analyzed the profile of m6A content in OS tissues. m6A methylation quantification showed that m6A content was decreased in OS tissues compared with adjacent normal tissues (Figure 3a). m6A methylation quantification and dot blot assays were also employed to detect the effect of FTO on m6A methylation in OS cells. As expected, the m6A level was down-regulated in OS cells after expressing FTO (Figure 3b,c). Western blotting showed that FTO decreased KLF3 expression in OS cells, and silencing FTO conferred by siRNA inversely increased KLF3 (Figure 3d), which were consistent with results from qPCR (Figure 3e). IHC staining supported the decreased expression of KLF3 in OS tissues compared with normal tissues (Figure 3f). A negative correlation between FTO and KLF3 was also observed in OS tissues (Figure 3g). Thus, we speculated that FTO could negatively regulate the expression of KLF3 in OS.

Relative m6A content was performed in OS and adjacent normal tissues using methylation quantification assay (a). m6A quantification and dot blot assays were employed to detect the effect of FTO on m6A methylation in OS cells (b-c). The effects of FTO knockdown or overexpression on the protein and mRNA levels were determined by western blotting and qRT-PCR (d-e). Representative images of IHC staining were used to analyze the protein levels of FTO in OS tissues adjacent and normal tissues (f). Correlation analysis of FTO and KLF3 mRNA expressions in OS tissues (Pearson correlation test (g). Me-RIP assay was performed to measure m6A enrichment on KLF3 mRNAs in OS cells after knocking down FTO (h). RNA stability assay was used to detect the mRNA stability of KLF3 after FTO overexpression in cells with the treatment of actinomycin D (0.2 mM) for the indicated time (i). Transfection efficiencies of YTHDF2 knockdown or overexpression were analyzed by qPCR in OS cells (j). YTHDF2 attenuated the mRNA and protein levels of KLF3 in OS cells (k-j). The effect of YTHDF2 on the mRNA stability of KLF3 was performed (m). RIP assay was performed to analyze the interaction between YTHDF2 protein and KLF3 mRNA in OS cells (n). The effect of silencing YTHDF2 on FTO-mediated the decrease of KLF3 mRNA and protein was performed by qRT-PCR and Western blotting (o-p). The role of FTO-induced mRNA degrading of KLF3 in OS cells was analyzed (q). Data represent the means ± SD. **, *P* < 0.01; ***, *P* < 0.001.

### 3.4 FTO regulates KLF3 expression in an m6A-dependent manner

The functional interaction between m6A methyltransferase and demethylase determines the dynamic and reversible regulation of m6A modification. And m6A readers bind to m6A-modified mRNA, thus affecting its fate [29]. Me-RIP assay suggested that knockdown of FTO augmented the m6A enrichment on KLF3 mRNAs in OS cells (Figure 3h). FTO decreased the expression of KLF3 mRNA (Figure 3e). mRNA stability assays indicated that, after treatment with transcription inhibitor Actinomycin D, the residual percentage of KLF3 mRNA in the FTO group was significantly lower than that in the control group (Figure 3i). Previous studies have confirmed that m6A binding protein YTHDF2 affects the stability of m6A-modified RNA by locating them at mRNA decay sites [29]. Transfection efficiencies of YTHDF2 knockdown or overexpression were analyzed by qPCR in OS cells (Figure 3j). YTHDF2 attenuated the mRNA and protein levels of KLF3 (Figure 3k,l). And YTHDF2 reduced the mRNA stability of KLF3 (Figure 3m). The RIP assays proved the interaction between YTHDF2 protein and KLF3 mRNA, which was enhanced in OS cells after expressing FTO (Figure 3n). Silencing YTHDF2 abrogated FTO-mediated the decrease of KLF3 mRNA and protein levels in OS cells (Figure 3o,p). qPCR results also verified that YTHDF2 knockdown reversed FTO-induced mRNA degrading of KLF3 (Figure 3q). Overall, Silencing FTO accelerated the YTHDF2-involved decay of KLF3 mRNA in OS cells.

### 3.5 KLF3 impairs the FTO-induced proliferation and invasion of OS cells

**Figure 4.**
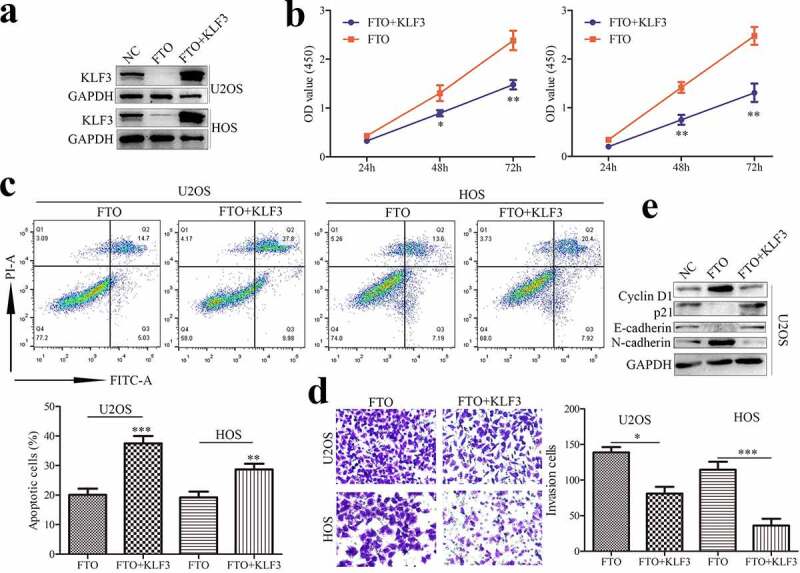
KLF3 impairs the FTO-induced proliferation and invasion of OS cells.

To ascertain whether KLF3 is involved in FTO function, OS cells transfected with FTO plasmids showed lower expression of KLF3, which could be reversed by overexpressing KLF3 (Figure 4a). Moreover, FTO promoted the proliferation of OS cells, whereas enforced expression of KLF3 reversed the FTO-induced promotion effects (Figure 4b). FTO inhibited apoptosis, which was severely impeded in OS cells with ectopic expression of KLF3 (Figure 4c). Analogously, KLF3 suppressed FTO-induced cell invasion (Figure 4d). Cyclin D1 and p21 are key proteins of cell cycle regulation. FTO increased Cyclin D1 expression and decreased p21 expression in OS cells (Figure 4e), and KLF3 could reverse the alternation. EMT also plays an important role in the process of tumor metastasis. FTO could enhance N-cadherin and Vimentin expression and suppress E-cadherin expression (Figure 4e), which were compromised by KLF3 amplification. Thus, our findings indicated that FTO promoted cell proliferation and invasion by inhibiting KLF3 expression in OS cells.

KLF3 could reverse the FTO-induced decrease of KLF3 protein (a). KLF3 impaired the FTO-induced promotion effect on OS cell proliferation (b). Flow cytometry assay was performed to investigate the role of KLF3 in FTO-involved apoptosis in OS cells (c). The effect of KLF3 on FTO-induced cell invasion (d). The effects of FTO and (or) KLF3 on the cycle regulator and EMT markers in OS cells (e). Data represent the means ± SD. **, *P* < 0.01; ***, *P* < 0.001.

## 4. Discussion

m6A methylation, as the most important RNA internal modification in eukaryotes, was first discovered in 1974 and attracted great attention recently [6,7]. m6A methylation occurs in all stages of the RNA life cycle, from RNA synthesis to nucleation, translation regulation to RNA degradation [29]. m6A modification is closely related to the occurrence and development of cancer and participates in cell differentiation, proliferation, migration, immunity, and drug resistance. Deregulation of m6A regulators in OS is inevitable [11,13,30,31]. Miao et al. reported that METTL3 contributes to proliferation, migration, and invasion of OS cells and activates the Wnt/β-catenin signaling pathway by regulating m6A modification of LEF1 mRNAs [11]. Analogously, METTL3 also promotes proliferation and invasion of OS cells by increasing ATAD2 expression via an m6A-dependent manner [12]. ALKBH5 attenuates the m6A modification of PVT1 mRNA and impedes the binding of YTHDF2 to PVT1 mRNA, which increases PVT1 expression and accelerates OS growth in vivo [13]. In addition, m6A-related enzymes are involved in regulating the pluripotency of OS stem cells [31,32]. All these imply potential roles of m6A methylation in tumorigenesis and progression of OS. And the relationship between FTO and OS has not been reported.

In the study, our results showed that the protein and mRNA levels of FTO were increased in OS tissues and cells. Through IHC staining, we also found that FTO expression in OS tissues was significantly higher than that in paracancerous normal tissues, and increased FTO was associated with poor prognosis and aggressive phenotypes in OS. Ectopic expression of FTO promoted the proliferation, migration, and invasion of OS cells and inhibited apoptosis, and knockdown of FTO shows the opposite effects. These findings supported that FTO promoted OS progression.

KLFs are one of the key components of the mammalian Sp/KLFSp zinc finger protein family. KLF3, as a member of KLFs, can recruit transcriptional corepressor C-terminal-binding protein to promoters of downstream molecules and primarily effectuate transcriptional inhibition [20,22]. Similar to other Sp/KLFSp zinc finger protein members, KLF3 contains conserved binding sites of GC-rich motif or CACCC box, which causes the regulation diversity for target genes [33]. Therefore, KLF3 has multiple biological functions, including erythropoiesis, adipogenesis, muscle regulation, B-cell development, differentiation, and apoptosis [22,34]. Aberrant expression of KLF3 is related to the occurrence and progress of various tumors, such as acute leukemia [22], sarcoma [24], lung cancer [21], colorectal cancer [23], and melanoma cancer [35]. Loss of KLF3 potentiates lung cancer metastasis and EMT process through controlling the STAT3 pathway [21]. DNA methylation-mediated KLF3 silencing augments the pro-metastatic miR-182 in human sarcoma cells [24]. Decreased KLF3 is associated with poor prognosis and may work as a promising predictor in colorectal cancer [23]. These suggest that KLF3 may be a potential tumor suppressor gene. It has been reported that m6A-mediated modification could regulate the methylation of other members of KLFs, such as KLF1 [36], KLF4 [37], and KLF5 [38]. RNA demethylation FTO promotes the phenotype conversion of human aortic vascular smooth muscle cells through increasing KLF5 expression in an m6A-dependent manner [38]. However, the potential mechanism of whether KLF3 is affected by m6A-mediated methylation has never been reported. Subsequently, we further explored whether FTO-mediated m6A modification regulates the expression of KLF3. We quantitatively detected the m6A modification in OS and adjacent tissues and found that m6A content in OS samples was lower than that in adjacent samples, It is consistent with previous studies that the total m6A level is decreased in OS [39]. After knocking down and overexpressing FTO, we detected the changes in KLF3 expression and found that FTO negatively regulated the mRNA and protein expressions of KLF3 in OS cells. IHC staining showed that KLF3 was down-regulated in OS tissues compared with normal tissues.

And decreasing FTO can potentiate m6A modification of KLF3 mRNA. As m6A ‘reader’, the C-terminal of YTHDF2 can specifically recognize m6A-modified mRNAs to reduce the stability of mRNAs [40]. We also proved the interaction between YTHDF2 protein and KLF3 mRNA in OS cells. Given that FTO negatively regulated the content of KLF3 mRNA, we investigated the effect of FTO-mediated m6A on the stability of KLF3. Our results showed that FTO decreased the stability of KLF3 mRNA, and deletion YTHDF2 impeded the decay of KLF3 mRNA induced by FTO. There was also a negative correlation between FTO and KLF3 expression in OS tissues. We concluded that FTO regulated the level of KLF3 mRNA in an m6A-dependent manner in OS.

In vitro studies demonstrated that FTO promoted the proliferation and invasion of OS cells. However, KLF3 protected against FTO-induced promotion effects on cell proliferation and invasion. Cyclin D1 promotes cell proliferation by binding to and activating cyclin-dependent kinase CDK4 unique to the G1 phase. And p21 can form a complex with cyclin D/CDK and arrest the cell cycle in the G1 phase. EMT concomitant with the down-regulation of epithelial markers and up-regulation of mesenchymal phenotype markers is an important process of invasion and distant metastasis of cancer. FTO up-regulated Cyclin D1 and N-cadherin levels and down-regulated p21 and E-cadherin levels in OS cells, and expressing KLF3 compromised the FTO-induced alternations. Collectively, FTO enhances cell proliferation and invasion by silencing KLF3 expression in OS cells. This study is the first to focus on the effects of FTO on the proliferation and invasion of OS cells by regulating the m6A methylation of KLF3 mRNA. Our results enrich the role of m6A modification in OS progression. However, further research is needed to provide direct epigenetic evidence of the FTO/KLF3 pathway in the pathogenesis of OS.

## 5. Conclusions

Collectively, this study reveals that FTO is overexpressed in OS and affects the prognosis survival of OS patients. FTO promotes the proliferation and invasion and inhibits apoptosis of OS cells through regulating KLF3 expression via an m6A-dependent manner. Our study provides a new direction for the molecular mechanism of OS progression by revealing the previously unknown linkage between FTO and KLF3. In addition, considering the importance of m6A modification in human cancers, it is possible to explore new strategies for the diagnosis and treatment of OS by targeting FTO with selective inhibitors.

## Supplementary Material

Supplemental Material

## Data Availability

All data and materials are available for verification as needed.
